# Shock Treatment: Using Immersive Digital Realism to Restage and Re-examine Milgram’s ‘Obedience to Authority’ Research

**DOI:** 10.1371/journal.pone.0109015

**Published:** 2015-03-02

**Authors:** S. Alexander Haslam, Stephen D. Reicher, Kathryn Millard

**Affiliations:** 1 School of Psychology, University of Queensland, Queensland, Australia; 2 School of Psychology and Neuroscience, University of St. Andrews, Fife, Scotland; 3 Department of Media Music Communication and Cultural Studies, Macquarie University, Sydney, New South Wales, Australia; University of Pécs Medical School, Hungary

## Abstract

Attempts to revisit Milgram’s ‘Obedience to Authority’ (OtA) paradigm present serious ethical challenges. In recent years new paradigms have been developed to circumvent these challenges but none involve using Milgram’s own procedures and asking naïve participants to deliver the maximum level of shock. This was achieved in the present research by using Immersive Digital Realism (IDR) to revisit the OtA paradigm. IDR is a dramatic method that involves a director collaborating with professional actors to develop characters, the strategic withholding of contextual information, and immersion in a real-world environment. 14 actors took part in an IDR study in which they were assigned to conditions that restaged Milgrams’s New Baseline (‘Coronary’) condition and four other variants. Post-experimental interviews also assessed participants’ identification with Experimenter and Learner. Participants’ behaviour closely resembled that observed in Milgram’s original research. In particular, this was evidenced by (a) all being willing to administer shocks greater than 150 volts, (b) near-universal refusal to continue after being told by the Experimenter that “you have no other choice, you must continue” (Milgram’s fourth prod and the one most resembling an order), and (c) a strong correlation between the maximum level of shock that participants administered and the mean maximum shock delivered in the corresponding variant in Milgram’s own research. Consistent with an engaged follower account, relative identification with the Experimenter (vs. the Learner) was also a good predictor of the maximum shock that participants administered.

## Introduction

I liked my psychotherapist, Dr Baum, but I had to argue with what she was trying to achieve. She wanted to cleanse the guilt I carried around with me, which would give me a start on dealing with the depression, and remove some of the inner conflict that was causing the anxiety. The quickest way out was to get me to deny responsibility.

People do terrible things during war. I was acting under orders. Had I heard of the Milgram experiment? Yes, I told her I had. (This surprised her.) This was the experiment where normal people were ordered to deliver shocks to someone behind a curtain. The shocks were not real and the screams were those of an actor, but the subject didn’t know that. Nevertheless, they continued to follow orders. “People go into a state of agency and act not on their own volition,” she explained to me. She made it sound like they didn’t have a choice, and I knew that was wrong.

Tony Lagouranis [1].

Stanley Milgram’s studies of ‘Obedience to Authority’ (OtA) can lay some claim to being the best known in the whole of psychology [2], [3], [4], [5]. In these, members of the New Haven community (mainly men) volunteered to participate in a study ostensibly designed to investigate the effects of punishment on learning. When they turned up at the lab, they found themselves cast in the role of a ‘Teacher’ and with the job of administering electric shocks to another man (the ‘Learner’) whenever he made an error on a word recognition task. The shocks were administered progressively via a machine on which 30 switches signified escalating levels of shock – starting at 15 volts (designated ‘slight shock’) but rising to 450 volts (ominously designated ‘xxx’).

In fact the machine did not deliver shocks and the Learner was a confederate, but the Teachers did not know this. The true purpose of the research was also not to study memory, but to see how far participants would go in following the Experimenter’s instructions. Most critically, would they be willing to administer a potentially lethal shock to an innocent man when instructed to do so by a figure of authority?

When Milgram asked ordinary people what they thought they would do, most believed that they would go no further than 135 volts. None believed that they would go above 300 volts, let alone all the way to the 450-volt maximum. When he asked psychiatrists what they thought ordinary people would do, they predicted that only a pathological fringe constituting some 5% of the population would go beyond 300 volts, and that only just over 0.1% would go to the maximum [6]. Yet when Milgram ran what he originally termed the “Coronary condition”, 26 out of 40 ordinary Americans (65%) went up to 450 volts. In this condition (which later became known as the “New Baseline” [7], [8]) the Learner reacts to the ‘shocks’ with a series of scripted exclamations and protestations, including, at 150 volts, “Ugh!!! Experimenter! That’s all. Get me out of here, please. My heart’s starting to bother me. I refuse to go on. Let me out.”, and at the 330-volt point he screamed “Let me out of here… My heart’s bothering me. Let me out, I tell you. (*Hysterically*.) Let me out of here.” [6], [9].

The fame of Milgram’s studies derives largely from the sheer power and unexpectedness of these results. Unlike most psychological experimentation, in which laboratory phenomena are far more pallid than their real-world counterparts, Milgram seemed to have reproduced in his studies the very types of horrific conduct that had scarred the history of his times. The parallel with the Nazi holocaust seemed direct and compelling. What is more, the impact of Milgram’s work was greatly enhanced by his work as a film-maker and the documentary *Obedience* was critical in bringing his findings before a wider public [10].

But besides the phenomena themselves, there are two other things for which the OtA experiments are remembered and which reverberate still. The first is conceptual and has to do with a shift from dispositionalism to situationism in the explanation of human behaviour. This relates to Milgram’s conclusion that “it is not so much the kind of person a man is as the kind of situation in which he finds himself that determines how he will act” [2] (p. 101) and that “the ordinary person who shocked the person did so out of a sense of obligation – a conception of his duties as a subject – and not from any peculiarly aggressive tendencies” [6] (pp. 23–24). More formally, these observations became the basis for Milgram’s *agentic state theory* which argued that participants were so focussed on the authority, and so bound up with the task of carrying out instructions to the best of their ability, that they lost sight of the consequences of their actions and of their moral implications. In the simpler terms used by the *New York Times* when announcing Milgram’s findings to the world, this means that people cannot help but “blindly obey orders” [11]. In coming to this conclusion, Milgram also acknowledged a debt to Hannah Arendt’s concept of the ‘banality of evil’ derived from her observations at the trial of the Nazi bureaucrat Adolf Eichmann [6], [12].

The second reason why the OtA studies are remembered is for the ethical controversy they generated. This controversy began almost immediately after the publication of Milgram’s first paper when Diana Baumrind wrote an article for *American Psychologist* in which she noted the extreme tension experienced by Milgram’s participants and decried “the kind of indignities to which [his] subjects were exposed” [13] (p. 423). She concluded: “I would not like to see experiments such as Milgram’s proceed unless the subjects were fully informed of the dangers of serious aftereffects and his correctives were clearly shown to be effective in restoring their state of well being” [13] (p. 423). In his defence, Milgram invoked post-experimental survey data and psychiatrists’ reports to demonstrate that most participants were glad to have participated and that none had been harmed [14].

Elsewhere we have argued that both Baumrind and Milgram miss a key point here. For even though Milgram managed to reconcile his participants to what they had done, it is apparent that he only achieved this by convincing them that it was acceptable to cause suffering in the name of scientific progress [15]. Nevertheless, however one argues the case, ethical concerns render it impossible to replicate Milgram’s study in today’s world. And this creates a dilemma. For if one wants to question the conceptual account used by Milgram to explain his findings, one is prevented from doing so by the impossibility of using his paradigm to examine exactly what factors do (or do not) produce obedience.

In a sense, then, the two things for which Milgram is remembered have become mutually sustaining in so far as the ethical controversy has made the theory more resilient. Indeed, this is a key reason why notions of the banality of evil and of the inherent blindness of obedience continue to dominate contemporary teaching and scholarship as well as popular thinking around these issues [16], [17]. There are, however growing reasons to think that these ideas are inadequate and hence for wanting to find a way to challenge their empirical basis.

## Questioning the Banality of Evil and the Blindness of Obedience

As we have already intimated, one of the reasons for Milgram’s impact has to do with the resonance between his studies and real-world phenomena – notably the Holocaust. Here Arendt’s portrayal of Adolf Eichmann as an ordinary bureaucrat, so focussed on making the trains run on time that he forgot he was transporting millions to their death, seemed to put the stamp of historical authenticity on Milgram’s analysis. Yet a range of recent studies of perpetrators in general, of Nazi functionaries, and of Eichmann himself have questioned just how banal and unaware these people were [18], [19], [20], [21]. Contrary to the suggestion that they were merely puppets of those in authority, it seems that they knew exactly what they were doing, that they believed in what they were doing, and that they showed considerable creativity in pursuing and exterminating their victims. In short, they were committed and creative disciples of a collective cause [22]. As the prosecutor, Gideon Hausner noted, this meant that Eichmann had “not only fulfilled orders in rounding up Jews for deportation to extermination camps, but had gone about his work with extraordinary zeal and initiative” [23].

But while evidence from history might begin to cast doubt on the agentic state account, perhaps the most compelling case against this model comes from close scrutiny of Milgram’s own findings. Three issues, in particular, are relevant here.

First, when one looks at the full range of experimental variants that Milgram conducted (as opposed to focusing only on the New Baseline condition), it is apparent that ‘obedience’ varied from 0% to 100%. What is more, this variation in obedience cannot be explained through variations in the extent to which they encouraged participants to cede responsibility to the Experimenter [24]. More generally, then, the agentic state model fails to engage with the fact that these are studies of *disobedience* as well as obedience.

Second, there is a range of evidence that points to the fact that participants pay heed to the Learner as well as the Experimenter. In particular, Packer [25] has shown that the points at which participants are most likely to break off from the study are those where the Learner utters his most vehement protests (notably the 150-volt mark). As Nick Haslam and colleagues conclude, these and similar findings imply that any model, such as the agentic state account, which “sees the study exclusively through the lens of the Experimenter’s influence on the Teacher … must be incomplete” [26] (p. 9). Teachers attend to *both* the Learner and the Experimenter, and the key question becomes when and why they attend to one voice (the Experimenter urging ‘continue’) rather than the other (the Learner pleading ‘stop’).

Third, an analysis of the verbal interactions between the Experimenter and the Learner gives valuable insights into what succeeded, and what failed, in securing obedience. In particular, it is instructive to see what happened when the Experimenter used a series of four pre-defined ‘prods’ to urge the Teacher to continue if he proved reluctance to proceed. The first of these was a simple “please continue”, the second “the experiment requires that you continue”, the third “it is absolutely essential that you continue” and the fourth “you have no other choice, you must continue”. As we and others have noted, of these, only the last is a clear order, the others being a combination of requests and justifications [27], [28]. And yet, on nearly every occasion that the fourth prod was used, participants responded by refusing to continue. This can be seen in the following examples [29]:

E: You have no other choice, Teacher, you must continue.
T: Yes I do have a choice. I’m not going to go ahead with it.
E: Then we’ll have to discontinue the experiment then.
T: I’m sorry.

*[refuses to continue]*

E: You have no other choice, you must go on.
T: Yes I have a choice.
E: That is, if you don’t continue we’ll have to discontinue the experiment.
T: Just cut it out, after all, he knows what he can stand. That’s my opinion and that’s where I’m going to stand on it.

*[refuses to continue]*

A similar pattern of responses to prods emerged from a more recent replication of Milgram’s studies by Burger [27], [30] in which ethical problems were side-stepped by only requiring participants to administer shocks of up to 150 volts. Here, every time the fourth prod was used, participants refused to continue. There is, however, an important confound with order here, as it might be the case that, having already refused three prods, participants were disinclined to respond positively to a fourth (whatever its content). However, in a study where the four different prods were manipulated between participants (so that different participants received different prods in separate conditions), the order-like fourth prod clearly proved least effective [15]. Whatever else they show, Milgram’s studies thus provide little evidence of people blindly obeying orders [31].

In order to explain these findings, Haslam and Reicher argue that orders fail to secure compliance because they disrupt the inclusive relationship between the Experimenter and the Teacher. When the Experimenter issues a request or a justification, it suggests that he and the Teacher are involved together as partners in a common enterprise. An order, by contrast, sets the Experimenter apart from and against the participant. This analysis also accords with other evidence that these researchers have drawn upon in developing an ‘engaged followership’ model of obedience [15], [31], [32], [33].

According to this model, whether the Teacher attends to the voice of the Experimenter or the Learner – and hence whether he shows obedience or disobedience – hinges upon his identification with both parties. More specifically, do participants identify with the science of the study, and with the Experimenter as a representative of that science (in which case they obey), or do they identify with the Learner as a fellow member of the general community (in which case they disobey)? As an initial test of the model, contemporary observers were provided with Milgram’s own descriptions of his experimental variants and asked to estimate the extent to which the features of each would lead them to identify with the Experimenter and the Learner [33]. As predicted, there was a strong positive correlation between estimated identification with the Experimenter (iE) and the level of obedience observed in a particular variant, as well as a strong negative correlation between identification with the Learner (iL) and obedience, and a strong positive correlation between relative identification (iE – iL) and obedience.

Although these various findings are consistent with the engaged follower model, they are of course highly constrained by the fact of being rooted in post-hoc estimates and retrospective reinterpretations of archival data. In particular, we are not able to examine directly the role of constructs like identification because Milgram did not measure them. More definitive support for the model – or indeed for any alternative to the agentic state approach – depends on being able to design new studies and collect new data. But here we come back to our core dilemma in which the conceptual need for new understanding is constrained by the empirical (because unethical) impossibility of revisiting the classic obedience studies. Unless we are able to solve this dilemma – to devise studies of obedience that are controlled, impactful and ethical – our theoretical understanding of a core social phenomenon will remain rooted in the past [16].

## Overcoming Ethical Barriers to Progress and Exploring Disobedience

We have already pointed to some of the ways in which researchers have sought to overcome the ethical limits to obedience research. In addition to mining the archives, one approach has been to replicate the basic paradigm but to stop before the point that people are asked to inflict apparently lethal shocks and hence both limiting stress in the study and potential harm after the study [27], [30]. A different approach has been to produce a structural analogue of the Milgram paradigm in which participants are asked to inflict (apparent) harm on a target in an escalating series of steps. However, to deal with the ethical concerns, the harm is never as great as in the original. Besides the example provided above, which involves attributing negative attributes to groups [28], others have asked participants to give destructive feedback to job applicants [34], to feed insects into a crushing machine [35], to persist at a tedious task [36], or to administer noise blasts [37]. Yet another approach, developed by Slater and colleagues, has been to reproduce the Milgram paradigm in a virtual reality environment where shocks are delivered to a life-like avatar [38]. Importantly, the researchers were able to demonstrate the validity of this method by showing that, despite the contrived nature of the set-up, participants’ behavioural and physiological responses were very similar to those reported by Milgram [6].

These various approaches are highly creative and all have helped to advance our understanding of the obedience process. They have also led to a resurgence of interest in Milgram’s work [39], [40]. Yet, they still lack one element which is crucial to the impact of Milgram’s studies. For, in different ways, the different types of study try to diminish the harm to participants (and hence the ethical concerns) by diminishing the harm they are required to inflict another person – either because the act is less intense or because the other person is less real. But, in so doing they diminish the drama and distance the behaviours inside the laboratory from the real-world phenomena outside and hence lose the very thing that made Milgram’s studies so compelling and so impactful. As a result, it has proved difficult for the findings from such studies to challenge Milgram’s claims.

This raises the question of whether it is possible to find a way of maintaining the drama of Milgram’s studies while diminishing the harm. Can we ensure that people are fully involved in highly consequential actions without these actions having distressing or enduring consequences for the self? What is more, can we record these actions in a way that makes them as impactful as Milgram’s original?

To answer these questions, in the present research we report a study that employs the methodology of *Immersive Digital Realism* (IDR) to restage Milgram’s OtA research. This method draws on the rich tradition of realist film theory and practice [41] and was initially developed by Millard [42] to restage Gamson’s famous sociological research into encounters with an unjust authority [43].

IDR involves six core steps. First, one-to-one workshops between a film director and actors are used to develop composite fictional characters that draw in part on selected aspects of the actors’ work histories and personal lives as well as those of their peer groups. Second, the method involves deep immersion in character and context in preparation for filming. Third, the director strategically withholds details of the dramatic context from the actors – so that, in the present instance, they are briefed to perform as participants in a social psychology experiment but not given any more specific information (e.g., about the nature of the study or its design). Fourth, the method uses real-world design and environments as much as possible. In the present case, this involved attempting to faithfully reproduce both the laboratory environment and the shock machine. Fifth, filming involves long takes and multi-camera coverage. This is made possible (and relatively inexpensive) by modern digital technologies and frees the actors to focus on their interactions with the environment and other participants. In the present case, individual takes or recordings were up to 45 minutes in length. Sixth, at the conclusion of the first take, the film director conducts a debrief with each actor providing information about the project and its aims, differentiating between the behavior of the character and how they personally might have behaved, and addressing any questions or concerns they might have.

In the present study, a seventh step was added to this protocol in light of the fact that IDR was also being used for the purpose of psychology research. This involved a second debrief by a psychologist that started by interviewing the actor to explore how they felt during the study and why they acted as they did, and collecting relevant psychometric data. After this, a full explanation of the social psychological aspects of the research was provided.

IDR can be seen as an extension of previous work which has examined issues raised by the Milgram paradigm using both role-playing techniques [4], [44], [45] and immersive video environments [46]. However, the critical difference lies in the use of professional actors who, guided by a professional director, are trained in the ability to embody a character and who then explore how that character would behave in context. That is, our participants are not acting *‘as if’* they were an individual in the Milgram paradigm but rather *‘as’* a character who is then put in the Milgram paradigm. In this sense the term ‘role play’ [47] is misleading in the context of IDR. What is more, IDR maintains the drama and intensity of the phenomena under consideration while also diminishing any harm due to this experience. Accordingly, it meets Baumrind’s [14] ethical criterion, but also meets our own criteria of doing so without promoting a pernicious ideology that ennobles the infliction of harm on others [15]. Indeed, a major part of the psychological debrief (Step 7 of IDR above) was to make participants aware of the dangers of such ideologies.

## The Present Study

The present study formed part of a larger trans-disciplinary project in which social psychology and documentary film researchers worked together to interrogate aspects of Milgram’s OtA studies. Indeed, in this regard, it is notable that Milgram [49] himself saw his studies as a combination of science and art – arguing that film is unique in providing researchers with richly textured records of human behaviour that can continually be reanalysed by other social scientists. The project was therefore designed so that separate film and social psychology studies would each dovetail into the other. Specifically, film researchers addressed significant gaps in the audio-visual records through the authoring of a contemporary film interrogating Milgram’s *Obedience* whilst social psychology researchers built on IDR to examine aspects of participant behaviour within the paradigm. This involved an extended period of preparation that used the Milgram archives and other materials to see exactly how the original work had been staged and recorded [10]. It also involved a lengthy period of dialogue between the film researchers and the psychologists so each could understand the basis of the others’ work.

In this paper, we focus on the social psychology study. In drawing on IDR to revisit the Milgram paradigm, this had two key goals. The first was to validate our core claim that IDR allows us to explore how people behave in extreme contexts such as the Milgram paradigm. In particular, given that participants are actors, it needs to be shown that their behaviour corresponds to the behavior actually seen in the original studies rather than to people’s beliefs about their likely behavior (Slater uses a similar logic to interrogate virtual reality methods [38]). As Milgram himself noted, these are very different things since people radically underestimate how far they will go in inflicting shocks (only 24% think that they would go beyond 150 V and none believe that they would go beyond 300 V). The key question, then, is whether in IDR the level of shock that participants administer is more akin to the behaviour of real participants or to the estimates of non-participants. Moreover, given that shock levels differ across variants of the Milgram paradigm, it is pertinent to ask whether IDR participants show similar variation in the level of shock that they are prepared to inflict. Finally, turning from levels of obedience to the interactions that underpin this, it is important to establish whether the prod from the Experimenter that most resembles an order (i.e., Prod 4) leads people to carry on shocking (as popularly understood) or to stop doing so (as is actually the case)?

To explore these questions, as well as recreating Milgram’s original *Coronary* (or New Baseline) condition (his Experiment 5 [6]), we also restaged four other variants designed to capture the full range of behaviour that the paradigm elicits [33]. These were those in which (a) there was no feedback from the Learner (Milgram’s original pilot; *No L Feedback*), (b) two confederates withdrew from the experiment, leaving the naive participant to continue alone (Milgram’s Experiment 17, *2 Peers Rebel*), (c) the Experimenter left the room and gave instructions from afar (Experiment 7, *E Absent*), and (d) the Learner was connected to the shock machine in the same room as the Teacher (Experiment 3, *L Proximal*).

In assessing whether IDR is able to capture the same behaviour as Milgram’s studies, the study tested three key hypotheses derived from the body of previous research discussed above:

H1. That a majority (rather than a minority) of participants in the IDR paradigm would prove willing to administer shocks greater than 150 volts.
H2. That the mean maximum level of shock delivered in original variants of the OtA paradigm (as reported by Milgram [6]) would predict the maximum level of shock administered by participants in different variants of the IDR paradigm.
H3. That when participants in the IDR paradigm are given Prod 4 as a means of urging them to continue, this would encourage them to *dis*continue.

To the extent that these hypotheses are supported, a second goal of the study was to use IDR to explore why people do (or do not) obey the Experimenter. More specifically, we sought to explore issues related to the engaged followership model [32], [33]. In the first instance this would be supported by evidence consistent with H3. However, to explore this in more detail, during post-experimental debriefing we also asked participants to indicate the extent to which, in the course of the study, they had (a) identified with the Experimenter and the scientific project he was leading and (b) identified with the Learner and the broader community of which he was a representative. This allowed us to test a further hypothesis:

H4. That the maximum level of shock administered by participants in the IDR paradigm would be predicted by their relative identification with the Experimenter (vs. the Learner).

## Methods

### Participants

Participants were 14 actors (8 men, 6 women) chosen to participate in the study on the basis of their proven competence as professional actors. A further 4 actors were recruited to play the role of confederates (1 as Experimenter, 1 as Learner, 2 as Teachers in the Two Peers Rebel condition). (An additional participant was assigned to the Bring-a Friend Condition discussed by Rochat and Blass [50] and Russell [51]). However, because we did not have access to sufficiently detailed information about how to recreate this condition, our operationalization of it failed and was aborted).

Prior to the study, the actors worked with a director (KM) to develop fictional characters that they would play in the study. This involved drawing on past biographical and professional experiences to create a *composite character*. Participants were told that their character would be taking part in a social psychology experiment for which they had volunteered. However, beyond this, they were given no specific details about what to expect when they turned up to participate in the study.

### Materials, design and procedure

**Figure 1:**
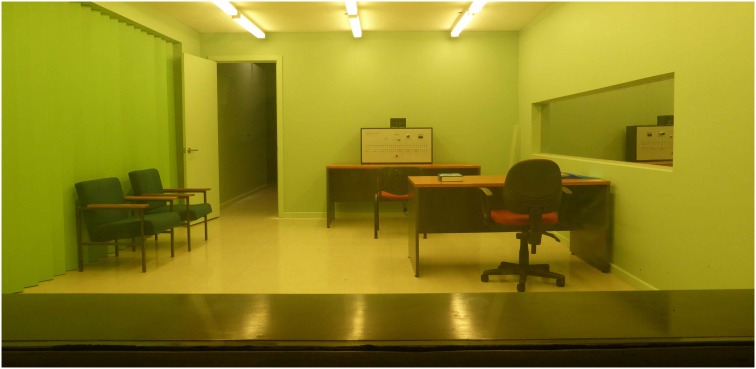
The set for the research laboratory.

As far as possible, the materials for the study were modelled on those developed by Milgram for his original studies (i.e., as described by Milgram [6], [9]). In particular, this involved building a laboratory set of the same size and with similar layout and furnishings as well as building similar apparatus (see Figure 1).

Three participants took part in the study on each of five consecutive days. They were randomly assigned to conditions, but conditions were run in an order that met the logistical demands of setting up the laboratory and briefing confederates on any given day. Due to its prominence as a reference point for Milgram’s work, half of the participants (i.e., 7) were assigned to the Coronary condition. Two further participants were assigned to No Learner Feedback, 2 Peers Rebel, and Experimenter Absent conditions and one to the Learner Proximal Condition^1^.

The procedure for running each condition was intended to replicate Milgram’s own procedure as closely as possible (see Milgram [6], [9] for details). Additional material was taken from the Milgram archives). In particular, this meant that in all variants other than the No Learner Feedback condition, the Learner made a series of pre-determined protests after receiving particular level of shock. When the Teacher proved unwilling to continue, he was also encouraged to do so using Milgram’s series of four escalating prods. It is worth noting, however, that as was the case in Milgram’s own research [3], [48], the requirements for the Experimenter to engage in meaningful discourse with the Teacher meant that it proved hard for him to stick rigidly to this script.

Participants were filmed using cameras and microphones concealed behind one-way glass and within the experimental apparatus. At the end of the study they were extensively debriefed: first, by the director (as part of the IDR process outlined above) and then by one of two psychologists (SAH or SDR). In the context of this debriefing they were also asked to respond on 11-point rating scales to four questions. Two assessed their character’s identification with the Experimenter and the Learner (as used by Reicher and colleagues [33]; Thinking back to the study, how much did you identify with the Experimenter and his scientific goals? Thinking back to the study, how much did you identify with the Learner as a member of the general community?; where 0 = did not identify at all, 5 = identified moderately, 10 = identified very much). Two assessed their stress during and after the study (as used by Milgram [15]; Thinking back to the point in the experiment when you felt most tense and nervous, how nervous did you feel? where 0 = not at all, 5 = moderately, 10 = extremely; All things considered, how do you feel about having participated in this study? where 0 = very sorry, 5 = neither sorry nor glad, 10 = very glad).

### Ethics statement

Participants were professional actors employed in that capacity by KM. Consent for the filmed components of the research took the form of a standard film industry written contract. At the conclusion of the director’s debrief, SAH and SDR interviewed participants and administered a short questionnaire to collect additional data. At this point additional verbal consent was obtained and recorded by SAH and SDR to use the data that had been collected for psychological research. Verbal consent was sought as it would have been impractical and disruptive to obtain written consent at this point in the procedure. All these arrangements were approved by the Human Research Ethics Committee at Macquarie University (the site of the study and the institution responsible for administering the main grant that supported the research; Approval No.: 5201300440). Approval was conditional on all outputs from the study making it clear that participants were actors. Additional approval was obtained from the Research Ethics Committees at the University of Queensland (Approval No.: 2014000828) and the University of St. Andrews (Approval No.: PS11050).

## Results

Data from the study are presented in Data S1.

### Tests of H1

Consistent with H1, a majority of participants administered shocks greater than 150 volts. Indeed, although there was considerable variability in their responses (SD = 89.5), all went as far as 195 volts (“very strong shock”) and, on average, they went as far as 300 volts (*M* = 301.1). This point is significantly greater than 150 volts (*t*(13) = 6.32, *p*<.001), but (non-significantly) lower than the weighted mean level of shock administered in the corresponding variants of Milgram’s studies (344.8; *t*(13) = −1.82, *p* = .09). It is also significantly greater than the midpoint (249.0) between the mean maximum shock that Milgram’s participants estimated they would deliver (146.4) and the mean maximum shock that they actually delivered (344.8; *t*(13) = 2.32, *p* = .04) – indicating that responses were closer to the latter than the former.

### Tests of H2

**Figure 2:**
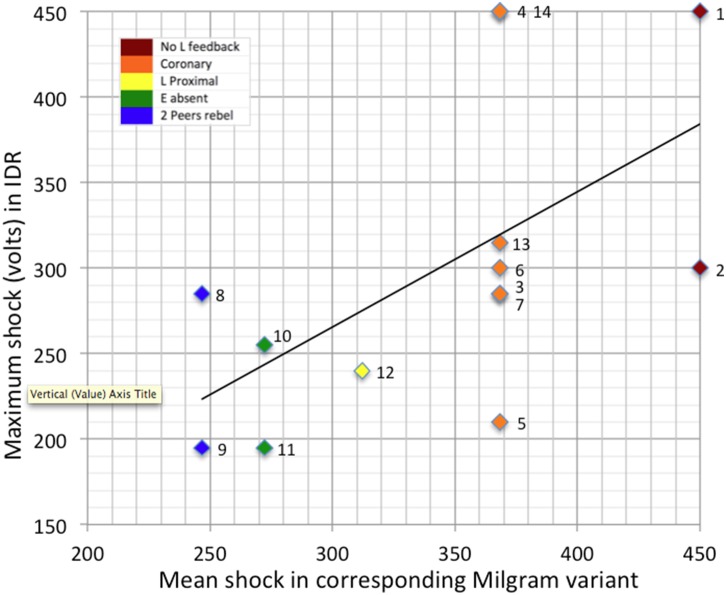
Scatterplot and regression line of maximum shocks delivered by participants in IDR study against mean maximum shocks in corresponding Milgram variant. *Note*: Numbers identify individual participants and correspond to the order in which they participated in the IDR study.

Figure 2 plots the maximum level of shock administered by participants in different variants of the IDR paradigm against the mean maximum level of shock delivered in the corresponding OtA variant (as reported by Milgram [6]). In line with H2, from this it can be seen that there was a positive linear correlation between these variables, such that the levels of shock observed in Milgram’s original variants were a strong and significant predictor of the maximum level of shock administered in the IDR paradigm (*r*(13) = .59, *p* = .03).

Qualitative data also pointed to a strong correspondence between the behaviour displayed in Milgram’s original studies [6], [49], [52] and that observed in the IDR paradigm. In particular, most participants were clearly agitated and stressed during the study itself, but also very relieved when it was concluded, as well as happy to have participated once debriefed [15], [16]. This was confirmed by post-experimental measures on which participants reported being tense and nervous during the study (*M* = 7.78; significantly greater than the scale midpoint, *t*(13) = 5.00, *p*<.001) but ultimately glad to have taken part in it (*M* = 7.07; significantly greater than the scale midpoint, *t*(13) = 3.52, *p*<.01).

### Tests of H3

Consistent with H3, it is apparent that of the ten participants who were given Prod 4 in an attempt to urge them to continue only one participant proceeded to administer further shocks while nine immediately discontinued (Binomial test, *p* = .01). Here too it is apparent that the manner in which participants broke off at this point closely resembled behaviour observed in Milgram’s original studies [29], [48]. In particular, they objected strongly to the Experimenter’s assertion that “you have no choice” and retorted that, in fact, they did (as also evidenced in other more recent studies [28], [30]). This is exemplified by the following exchanges:

### P3 Tom [Coronary]

E: It’s absolutely essential that you continue.
T: Essential to what?
E: Look you don’t have a choice, you need to …
T: Everybody’s got a choice, mate. Everybody’s got a choice. Like, like…you know this this guy’s in pain in here.
E: Are you refusing to go on?
T: Well, I’m uncomfortable to go on, yeah. He scr- have a listen to him.

*[refuses to continue]*

### P10 Lana [E absent]

E: Lana, it’s absolutely essential that you carry on.
T: Ok well you, you might have to do this with somebody else because I’m hearing somebody wanting to be let out I’m. I can’t …
E: I’m sorry, Lana, you don’t have a choice.
T: Sorry, what do you mean that I don’t have a choice?
E: You need to carry on.
T: I know that I didn’t understand what exactly I was signing up for but I feel very very uncomfortable.

*[refuses to continue]*

### P11 Helena [E absent]

E: It’s absolutely essential that you continue, Helena
T: Why is it essential? It’s essential that I hurt him, I don’t think so. James, are you alright?
E: Listen Helena
T: What?
E: You don’t have a choice …
T: I th-
E: … please continue
T: I think I do have a choice, John. And I choose not to continue.

*[refuses to continue]*

### Tests of H4

Consistent with observations that the drama of the Milgram paradigm derives from the fact that it creates a situation in which participants attend to both the Experimenter and the Learner, and hence are torn between the contradictory demands made of them by these two sources, in their post-experimental responses participants reported having high levels of identification with both the Experimenter (*M* = 6.64; significantly greater than the scale midpoint, *t*(13) = 2.24, *p* = .04) and with the Learner (*M* = 7.04; significantly greater than the scale midpoint, *t*(13) = 3.08, *p* = .01).

**Figure 3:**
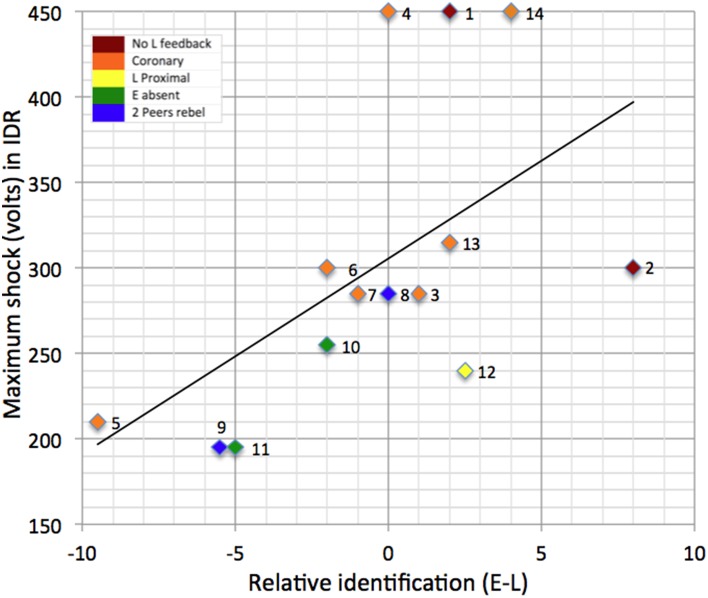
Scatterplot and regression line of maximum shocks delivered by participants in IDR study against relative identification with the Experimenter (versus the Learner). *Note*: Numbers identify individual participants and correspond to the order in which they participated in the IDR study.

In line with H4, it is also apparent that *relative* identification with the Experimenter and the Learner (i.e., iE – iL) determines which voice people attend to and hence their degree of obedience to experimental instructions. Thus relative identification was a good predictor of the maximum level of shock that participants were prepared to administer (*r*(13) = .56, *p* = .04). This can be seen from Figure 3 which presents a scatterplot and regression line for these data. Interestingly, though, while, on its own, identification with the Learner was a strong and significant negative predictor of the maximum shock delivered (*r*(13) = −.59, *p* = .03, identification with the Experimenter was only a moderate (but non-significant) positive predictor (*r*(13) = .36, *p* = .21).

## Discussion

The primary goal of this research was to explore the possibility of using a new methodology – Immersive Digital Realism (IDR) – to restage Milgram’s controversial Obedience to Authority (OtA) research in a way that is both impactful and ethical. While a number of paradigms have recently been developed for this purpose, key problems with these are that, in circumventing the ethical challenges of this task, they either do not involve administering the maximum level of shock [30], do not involve real participants [38], or do not recreate the dramatic tension of competing ties to Experimenter and Learner [28]. IDR addresses these problems by using actors who are naïve to the nature and purpose of the experimental paradigm to play the part of normal research participants. It uses trained participants who can both assume a character, as directed, during the study, while maintaining a clear separation between that character and their selves outside the study. In this way participation involves no harm for the actor.

Analytically, then, using this methodology raises two key questions: first, whether or not IDR does actually capture similar behaviour to Milgram’s original studies; second, whether or not it is capable of shedding light on the psychology of Milgram’s participants.

Speaking to the first of these questions, it is apparent from our quantitative analyses that there is a close correspondence between the behaviour observed in our IDR study and that observed in Milgram’s original research. This is evidenced in at least three ways. First, by the fact that participants, on average, went as far as 300 volts (much higher than people imagine they will, and only marginally lower than the levels found by Milgram). Second, by the fact that levels of obedience vary across conditions in the same way as in Milgram’s own studies. Third, by the fact that people overwhelmingly respond to orders (i.e. Prod 4: “You have no other choice, you must continue”) by disobeying rather than obeying (again, reflecting what people do in Milgram’s studies rather than what they believe that people do [27], [28]).

What is particularly interesting in relation to this last finding is that participants showed neither blind obedience (of the form the OtA studies are often understood to show [16]) nor reflexive disobedience (of the form people imagine themselves and others showing). Instead, resistance developed in response to the Experimenter’s attempt to deny participants’ sense of free will [53] and an associated violation of norms associated with shared identity (in which cooperation is understood to be voluntary rather than coerced [54]).

In addition to the statistical analyses we have provided, there are a number of other telling parallels between the behaviour of IDR participants and the behaviour of Milgram’s participants – as can be gleaned both from his filmed materials [52] and from recordings in the archives. For instance, it is clear that in both cases, participants are concerned with both the success of the experiment and the welfare of the Learner. They employ a range of strategies to try and overcome the contradiction between the two: even as they continue shocking they try to signal the right answer by pronouncing it more loudly, they show despair when the answer is wrong, they try to make the shocks as short as possible, they implore the Experimenter to check up on the Learner, and they try themselves to talk directly to the Learner and assess his welfare. Moreover, they are highly stressed by the contradictory demands put on them by the Experimenter and the Learner. This is clear in their behavior and it is also clear from their responses to psychometric measures during the psychological debriefing. Finally, and once more akin to the reactions of Milgram’s own participants, they show great relief when they meet the Learner and discover that he is unharmed [6].

At the same time – and this is the core of our ethical argument – there was a clear discontinuity between the actors and the characters they played. While participants acknowledged during both debriefings that the experience had been intense, none showed any sign of distress and all indicated that they had found the experience enlightening. Indeed, this ability to separate from one’s character is hardly surprising since it is, of course, a staple of the acting profession. In sum, our findings sustain the argument that IDR provides a means of exploring the full intensity of the Milgram paradigm in a way that gains ethical legitimacy without losing drama.

All this evidence together also begins to address the study’s second goal of investigating why Milgram’s participants acted as they did. In the first instance, it scotches suggestions that participants only attend to the Experimenter and ignore the Learner, as proposed in Milgram’s agentic state account. Clearly they attend to both and hence the key question becomes which voice takes precedence [26], [33]. Speaking to this point, it also confirms that orders do not increase the weight of the Experimenter’s voice, but rather diminish it. Moreover, it is apparent from the occasions on which Prod 4 was issued that orders disrupt the relationship between Teacher and Experimenter and lead the former seeking to assert their autonomy from the latter. This accords with an engaged follower perspective that sees obedience (vs. disobedience) as a function of the extent to which the Teacher identifies with the Experimenter over the Learner.

However, we also have more direct evidence to support this perspective. On the one hand, participants in character indicate a high level of identification with both Experimenter and Learner, thus confirming our contention that the Milgram paradigm is fundamentally dilemmatic [10]. On the other hand, levels of obedience (and differences in obedience between different variants of the paradigm) are predicted by relative identification as measured through post-experimental measures. This accords both with our own re-analyses of Milgram’s findings [33] and with those of others [26].

## Limitations and Future Research

Notwithstanding its advantages relative to other methods that have recently been developed to reopen the investigation of Milgram’s classic studies, it is clear that the present study also has some significant limitations. We have already discussed at some length obvious issues raised by the fact that participants were recruited as professional actors who were taking part in a staged production rather than as members of the community contributing to scientific research. Here again, though, we see the particular value of IDR as a research (and filmic) tool is that rather than immersing actors in a script, it immerses them in a character and then places them in a strong context in ways that allow for an in-depth exploration of the dynamic interaction between these elements (which is also why the professionalism of both actors and director is crucial). Because this interaction is central to the issues that are explored in social psychology – especially in its classic studies [55], [56] – we therefore see the method as compatible with the discipline’s core goals rather than at odds with them. Indeed, it is this dramatic staging that makes the discipline’s classic studies so compelling, not only as demonstrations of social psychological processes but also as film [10], [57], [58].

Nonetheless, IDR does raise serious issues that need to be acknowledged. Not least, combining documentary film using professional directors and professional actors with a social psychology experiment leads to logistical and financial constraints. It requires a lengthy period of set up, it requires extensive dialogue between the film makers and the psychologists so that each understands the perspectives and the requirements of the other. It requires filming facilities and often (as in this case) the construction of a set. There are limited windows in which it is possible to have the services of actors and each trial is lengthy, so that it is very difficult to run large numbers of participants. In the present study, this meant that our quantitative analyses all had relatively low power. This problem was to some extent mitigated by the fact that our hypotheses were clearly grounded in previous theory and research, and generally received strong support. However, if only to allow for robust statistical comparison of different experimental treatments, it would certainly be valuable (if expensive) for future research to involve a larger sample of participants.

## Concluding Comment

Since Milgram’s work was first published over half a century ago, researchers have been held in the grip of a powerful dilemma. On the one hand, Milgram uncovered a phenomenon of “great consequence” that they were impelled to study further [2]. On the other hand, the great controversy that his paradigm fuelled precluded them using his own procedures to do so [4]. In recent years researchers have used their ingenuity to resolve this dilemma by endeavouring to come as close as possible to the fire of the OtA paradigm without burning themselves on its ethical flames [31].

The present research represents a novel attempt to contribute to this collective effort.

As with any stand-alone piece of research, its has limitations. We are certainly not suggesting that IDR supplants the many other ways in which obedience is currently being studied. Nevertheless, as part of a rapidly growing corpus of work, we believe the study can add an essential element that other methods cannot supply. Most particularly, this is because it provides dramatic evidence of participants’ willingness not only to comply with an authority but also to resist it [59]. It also supports claims that the path they take is not pursued blindly, but follows lawfully from their relative identification with competing sources of influence [33].

In this too, the study provides clear evidence to support claims – like those of Tony Lagouranis, quoted at the start [1] – that obedience is not an ineluctable proclivity but a *choice*. Interestingly, the force of this point is seen most clearly when agents of influence attempt to deny their subordinates the opportunity to exercise free will [53]. For rather than strengthening compliance, this instead engenders resistance. Far, then, from showing that the landscape of tyranny is bereft of human agency, we see instead that identity-based choice is what makes tyranny possible – and also what makes tyranny vulnerable to overthrow. In these terms, as Lagouranis suggests, it is time to reject the comforts of the obedience alibi [20]. It is time instead, to engage with the uncomfortable truth that, when people inflict harm to others, they often do so wittingly and willingly.

## Supporting Information

Data S1 **Milgram IDR data file.**
(XLSX)
